# Supplementary material for: Diversity and Biological Activities of Endophytic Fungi from the Flowers of the Medicinal Plant Vernonia anthelmintica
Source: Int J Mol Sci. 2022 Oct 8;23(19):11935. doi: 10.3390/ijms231911935 (PMC9569571; doi:10.3390/ijms231911935)
Supplement: Supplementary file 1 [file ijms-23-11935-s001.zip › ijms-1908157-supplementary.pdf]

SUPPORTING INFORMATION

Diversity and biological activities of endophytic fungi from the flowers of the medicinal plant *Vernonia anthelmintica*

Niu Litao [1], Nigora Rustamova [1,2], Hui Xia Ning [1], Paiziliya Paerhati [1] and Abulimiti Yili [1]*

[1] Key Laboratory of Plant Resources and Chemistry in Arid Regions, Xinjiang Technical Institute of Physics and Chemistry, Chinese Academy of Sciences, South Beijing Road 40-Urumqi, Xinjiang 830011, PR China;
[2] Department of Biology, Samarkand State University, University Boulevard 15, Samarkand 703004, Uzbekistan.
* Correspondence: Correspondence: abu@ms.xjb.ac.cn; Tel.: +86-991-3835679 (A.Y.)

Abstract: Secondary metabolites produced by endophytic fungi are an important source of biologically active compounds. The current research was focused on the biological activities of ethyl acetate extracts of fungi, isolated and identified from *Vernonia anthelmintica* flowers for the first time. In addition, an investigation of the non-polar chemical composition of dichloromethane-ethyl acetate extracts of the most active fungal strain was carried out. The isolates included *Ovatospora senegalensis* NR-03, *Chaetomium globosum* NR-04, *Thielavia subthermophila* NR-06, *Aspergillus calidoustus* NR-10, *Aspergillus keveii* XJF-23 and *Aspergillus terreus* XJF-3 species. Strains were identified by 18S rRNA gene sequencinges methods and were registered in GenBank. Crude extracts of the fungi displayed *in vitro* biological activities including antimicrobial and cytotoxic activities. A melanin content assay was performed on murine B16 cells. An ethyl acetate extract of *O. senegalensis* NR-03 showed high anticancer and antimicrobial activity; therefore, we also studied the non-polar chemical composition of the dichloromethane-ethyl acetate fraction and identified 52 non-polar compounds with the different medium. This investigation discovered that the secondary metabolites of the total extract of endophytic fungi could be a potential source of alternative natural antimicrobial, cytotoxic and melanin synthesis activity in their host plant, and the isolation of bioactive metabolites may provide a lead to new compounds of pharmaceutical significance.

Keywords: *Vernonia anthelmintica* flowers; biological activities; non-polar chemical composition; endophytic fungi

Gene sequences of endophytic fungi

Aspergillus terreus XJF-3

ATTGATATGCTTAAGTTCAGCGGGTATCCCTACCTGATCCGAGGTCAACCTGGAAAAAAACAAGTTGCAAATAAATGCGTCGGCGGGCGCCGGCCGGGCCTACGGAGCGG
AAGACGAAGCCCCATACGCTCGAGGACCGGACGCGGTGCCGCCGCTGCCTTTCGGGCCCGTCCCCCGGGAGCCGGGGGACGAGGGCCCAACACACAAGCCGGGCTTGAG
GGCAGCAATGACGCTCGGACAGGCATGCCCCCCGGAATACCAGGGGGCGCAATGTGCGTTCAAAGACTCGATGATTCACTGAATTCTGCAATTCACATTAGTTATCGCATT
TCGCTGCGTTCTTCATCGATGCCGGAACCAAGAGATCCATTGTTGAAAGTTTTAACTGATTGCAAAGAATCACACTCAGACTGCAAGCTTTCAGAACAGGGTTCATGTTGGG
GTCTCCGGCGGGCACGGGCCCGGGGGCGAGTCGCCCCCCGGCGGCCAGCAACGCTGGCGGGCCCGCCGAAGCAACAAGGTACAATAGTCACGGGTGGGAGGTTGGGCCA
TAAAGACCCGCACTCGGTAATGATCCTTCCGCAG

Ovatospora senegalensis NR-03

AAAGCGGAGGAAAAGAAACCAACAGGGATTGCCCCAGTAACGGCGAGTGAAGCGGCAACAGCTCAAATTTGAAATCTGGCTTCGGCCCGAGTTGTAATTTGTAGAGGATG
CTTTGGGCGCGGCGCCTTCTGAGTTCCCTGGAACGGGACGCCACAGAGGGTGAGAGCCCCGTATAGTTGGACGCCTAGCCTGTGTAAAGCTCCTTCGACGAGTCGAGTAGT
TTGGGAATGCTGCTCAAAATGGGAGGTAAATTTCTTCTAAAGCTAAATACCGGCCAGAGACCGATAGCGCACAAGTAGAGTGATCGAAAGATGAAAAGCACTTTGAAAAG
AGGGTTAAATAGCACGTGAAATTGTTGAAAGGGAAGCGCTTGTGACCAGACTTGCGCCGGGCCGATCATCCGGGGTTCTCCCCGGTGCACTCGGCCCGGCTCGGGCCAGCA

TCGGTTCTCGCGGGGGGACAAAGGCCTCGGGAATGTAGCTCCTCCGGGAGTGTTATAGCCCGGGGCGTAATGCCCTCGCGGGGACCGAGGTACGCGCATCTGCAAGGATG
CTGGCGTAATGGTCACCAGCGACCCGTCTTG

Chaetomium globosum NR-04

CTGCGGAGGGATCATTACAGAGTTGCAAAACTCCCAAACCATTGTGGACGAACCTGACAGTTGCTTCGGCAGGTCGGCTTCCCGCCGCGCGGTGGCTCCTTGTGAGCCCGC
CTGCCGGAGTTATATAAACTCTTGATTATTGTTGACATCTCTGAGTAATTTACTTAATAAGTTAAAACTTTCAACAACGGATCTCTTGGTTCTGGCATCGATGAAGAACGCAG
CGAAATGCGATAAGTAATGTGAATTGCAGAATTCAGTGAATCATCGAATCTTTGAACGCACATTGCGCCCGCCAGTATTCTGGCGGGCATGCCTGTTCGAGCGTCATTTCAA
CCATCAAGCTTTAAGCTTGTGTTGGAGCTCTGCGGCTGTCCGCAGGCCCCTAAATACAGTGGCGGGCTCGCTATCACACCGAGTGCAGTAGTTTTCTTTCTCGCTCAGGGCGT
GTAGTGGGTGCTAGCCGTGAAACCCCTTTTTCACAAGGTTGACCTCGGATCAGGTAGGAATACCCGCTGAACTTAAGCATATCAA

Thielavia subthermophila NR-06

CTGCGGAGGGATCATTACAGAGTTGCAAAACTCCCTAAACCATCGTGAACGTTACCCCACAATCGTTGCTTCGGCGGGCGGCGCCTCACCCGGCGCCCCCTGCCAGGGGCC
CTAACGGGCGTCCGCCGGAGGCCTATAACCCTTGTATTATTTTACGGCTTCTCTGAGTTTATACTTAAATAAGTCAAAACTTTCAACAACGGATCTCTTGGTTCTGGCATCGA
TGAAGAACGCAGCGAAATGCGATAAGTAATGTGAATTGCAGAATTCAGTGAATCATCGAATCTTTGAACGCACATTGCGCCCCCCAGTATTCTGGAGGGCATGCCTGTTCG
AGCGTCATTTCAACCATCAAGCCCCGGGCTTGTGTTGGGGACCTGCGGCTGCCGCAGGCCCCGAAAAGCAGTGGCGGGCTCGCTGTCACACCGAGCGTAGTAGCAATCACC
TCGCTCAGGGCGTGCTGCGGGTGCCGGCCGTTAAACCCCCTTAACTTCTAAGGTTGACCTCGGATCAGGTAGGAGTACCCGCTGAACTTAAGCATATCA

Aspergillus calidoustus NR-10

TGCGGAAGGATCATTACCGAGTGCAGGTCTGCCCCCGGGCAGGCCTAACCTCCCACCCGTGAATACCTGACCAACGTTGCTTCGGCGGTGCGCCCCCCCCCGGGGGTAGCC
GCCGGAGACCACACCGAACCTCCTGTCTTTAGTGTTGTCTGAGCTTGATAGCAAACCTATTAAAACTTTCAACAATGGATCTCTTGGTTCCGGCATCGATGAAGAACGCAGC
GAACTGCGATAAGTAATGTGAATTGCAGAATTCAGTGAATCATCGAGTCTTTGAACGCACATTGCGCCCCCTGGCATTCCGGGGGGCATGCCTGTCCGAGCGTCATTGCTGC
CCTTCAAGCCCGGCTTGTGTGTTGGGTCGTCGTCCCCCCCGGGGGACGGGCCCGAAAGGCAGCGGCGGCACCGCGTCCGGTCCTCGAGCGTATGGGGCTTTGTCACCCGCTC
GATTAGGGCCGGCCGGGCGCCAGCCGGCGTCTCCAACCTTCTATTTTACCAGGTTGACCTCGGATCAGGTAGGGATACCCGCTGAACTTAAGCATATCA

Aspergillus keveii XJF-23

TGCATGTCTAGTATAAGCaATCTATACTGTGAAACTGCGAATGGCTCATTAAATCAGTTATCGTTTATTTGATAGTACCTTACTACATGGATACCTGTGGTAATTCTAGAGCTA
ATACATGCTAAAAACCCCGACTTCGGGAGGGGTGTATTTATTAGATAAAAAACCAATGCCCCTCGGGGCTCCTTGGTGATTCATAATAACTTAACGAATCGCATGGCCTTGC
GCCGGCGATGGTTCATTCAAATTTCTGCCCTATCAACTTTCGATGGTAGGATAGTGGCCTACCATGGTGGCAACGGGTAACGGGGAATTAGGGTTCGATTCCGGAGAGGGA
GCCTGAGAAACGGCTACCACATCCAAGGAAGGCAGCAGGCGCGCAAATTACCCAATCCCGACACGGGGAGGTAGTGACAATAAATACTGATACGGGGCTCTTTTGGGTCT
CGTAATTGGAATGAGTACAATCTAAACCCCTTAACGAGGAACAATTGGAGGGCAAGTCTGGTGCCAGCAGCCGCGGTAATTCCAGCTCCAATAGCGTATATTAAAGTTGTT
GCAGTTAAAAAGCTCGTAGTTGAACCTTGGGTCTGGCTGGCCGGTCCGCCTCACCGCGAGTACTGGTCCGGCTGGACCTTTCCTTCTGGGGAAGCCCATGGCCTTCACTGGC
TGTGGGGGGAACCAGGACTTTTACTGTGAAAAAATTAGAGTGTTCAAAGCAGGCCTTTGCTCGGATACATTAGCATGGAATAATAGAATAGGACGTGCGGTTCTATTTTGTT
GGTTTCTAGGACCGCCGTAATGATTAATAGGGATAGTCGGGGGCGTCAGTATTCAGCTGTCAGAGGTGAAATTCTTGGATTTGCTGAAGACTAACTACTGCGAAAGCATTCG
CCAAGGATGTTTTCATTAATCAGGGAACGAAAGTTAGGGGATCGAAGACGATCAGATACCGTCGTAGTCTTAACCATAAACTATGCCGACTAGGGATCGGGCGGCGTTTCT
ATGATGACCCGCTCGGCACCTTACGAGAAATCAAAGTTTTTGGGTTCTGGGGGGAGTATGGTCGCAAGGCTGAAACTTAAAGAAATTGACGGAAGGGCACCACAAGGCGT
GGAGCCTGCGGCTTAATTTGACTCAACACGGGGAAACTCACCAGGTCCAGACAAAATAAGGATTGACAGATTGAGAGCTCTTTCTTGATCTTTTGGATGGTGGTGCATGGCC
GTTCTTAGTTGGTGGAGTGATTTGTCTGCTTAATTGCGATAACGAACGAGACCTCGGCCCTTAAATAGCCCGGTCCGCGTCTGCGGGCCGCTGGCTTCTTAGGGGGACTATCG
GCTCAAGCCGATGGAAGTGCGCGGCAATAACAGGTCTGTGATGCCCTTAGATGTTCTGGGCCGCACGCGCGCTACACTGACAGGGCCAGCGAGTACATCACCTTGGCCGAG
AGGCCTGGGTAATCTTGTTAAACCCTGTCGTGCTGGGGATAGAGCATTGCAATTATTGCTCTTCAACGAGGAATGCCTAGTAGGCACGAGTCATCAGCTCGTGCCGATTACG
TCCCTGCCCTTTGTACACACCGCCCGTCGCTACTACCGATTGAATGGCTCGGTGAGGCCTCCGGACTGGCGCAGGAGGGTTGGCAACGACCCCCCCGCGCCGGAAAGCTGG
TCAAACCCGGTCATTAGAGAA

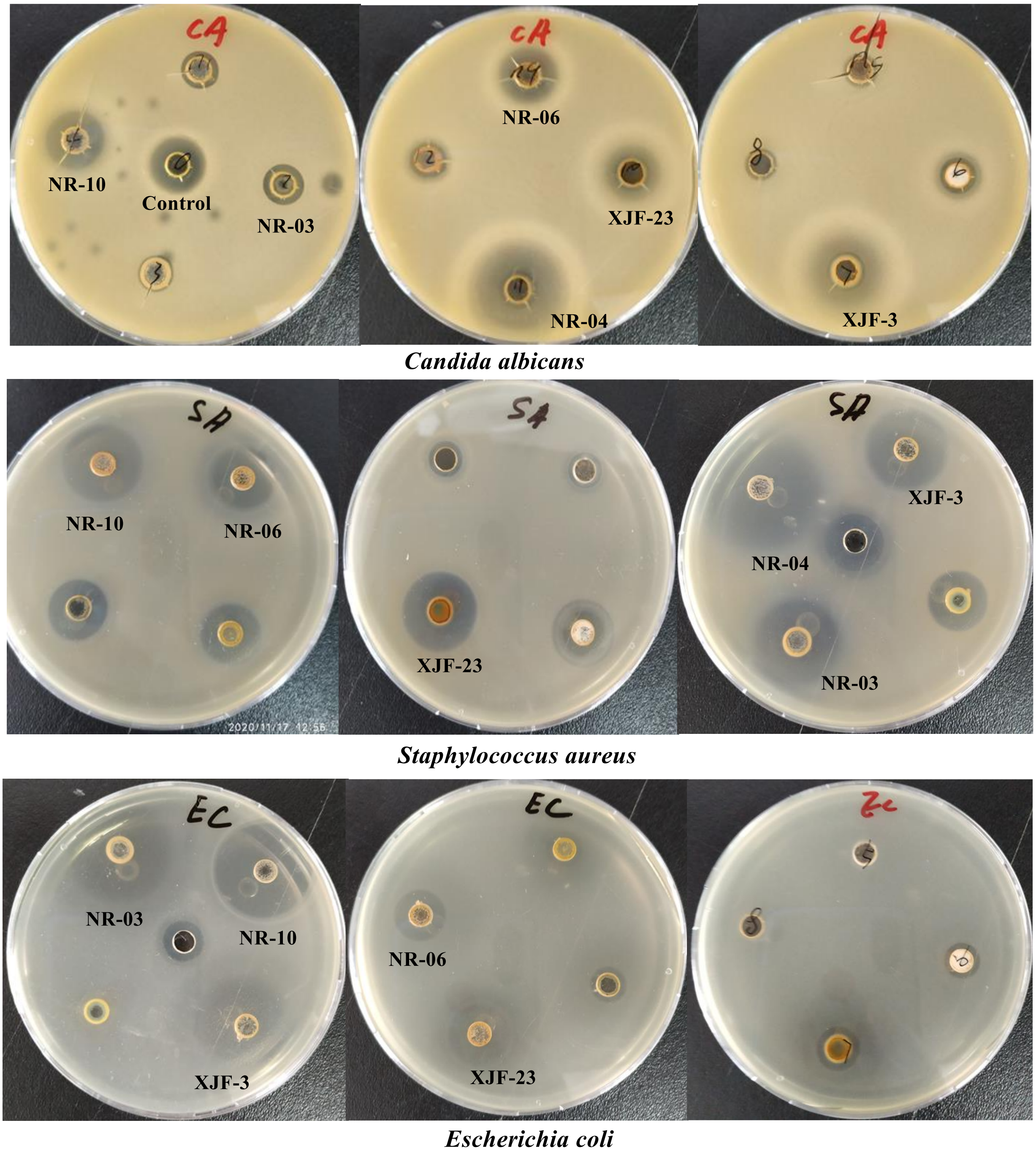

Figure S1. Antimicrobial activity of ethyl acetate crude extract of endophytic fungi evaluated by the diameter of inhibition zone (mm).

HPLC Analysis of secondary metabolites produced in different medium from endophytic fungus *Ovatospora senegalensis*

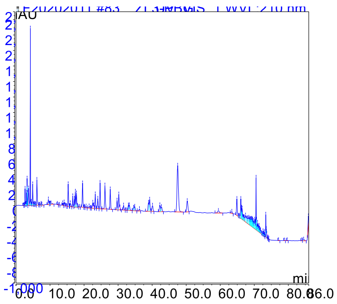

Figure S2. HPLC analysis of secondary metabolites produced by *O. senegalensis* NR-03 in the peptone beef extract glycogen medium (PBG)

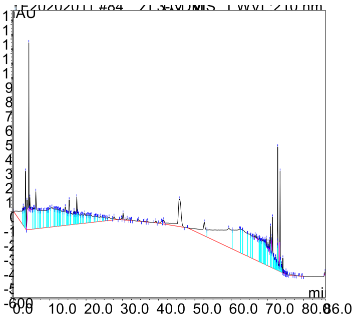

Figure S3. HPLC analysis of secondary metabolites produced by *O. senegalensis* NR-03 in the Czapeck-Dox medium (CDM)

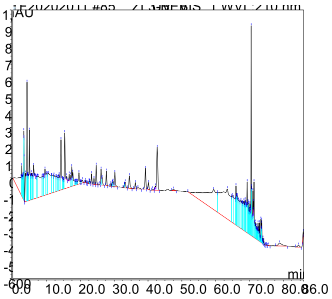

Figure S4. HPLC analysis of secondary metabolites produced by *O. senegalensis* NR-03 in the Beef extract broth (BEB)

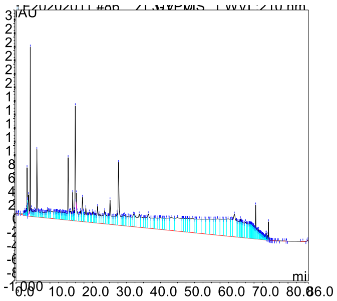

Figure S5. HPLC analysis of secondary metabolites produced by *O. senegalensis* NR-03 in the Yeast extract peptone dextrose (YPD)

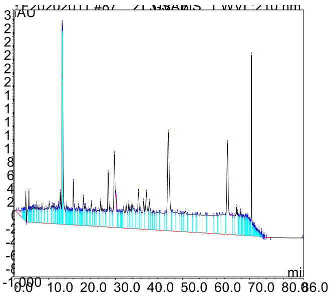

Figure S6. HPLC analysis of secondary metabolites produced by *O. senegalensis* NR-03 in the Sapouraund broth (SAB)

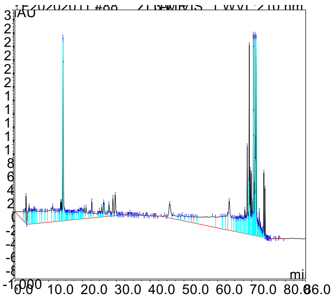

Figure S7. HPLC analysis of secondary metabolites produced by *O. senegalensis* NR-03 in the Malt extract broth (MEB)

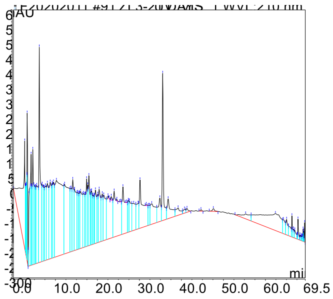

Figure S8. HPLC analysis of secondary metabolites produced by *O. senegalensis* NR-03 in the Potato Dextrose broth (PDB)

Chemical composition of endophytic fungus *O. senegalensis* NR-03 of analysed by GCMS

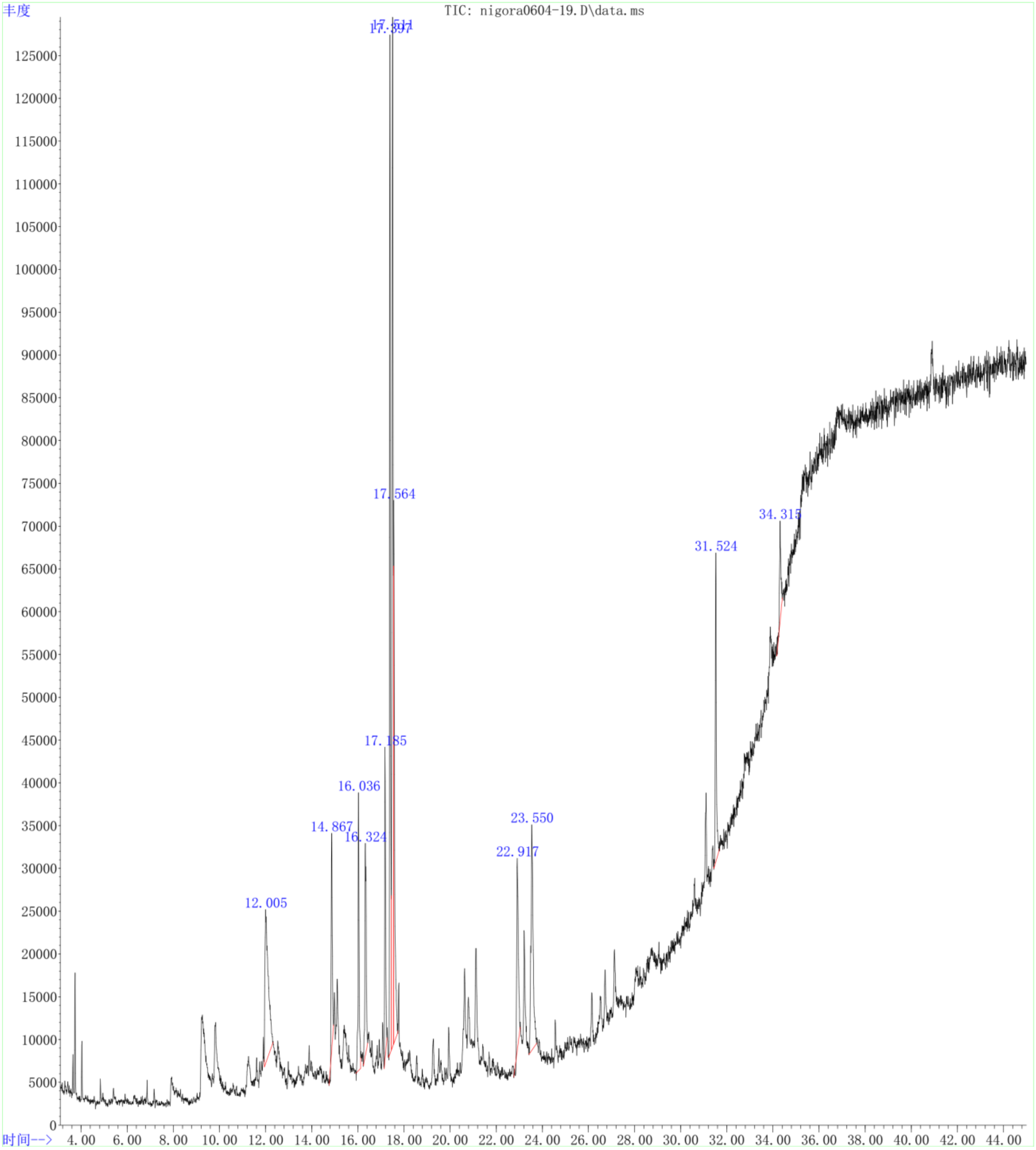

Figure S9. Volatile chemical composition dichloromethane extract of the endophyic fungus of *O. senegalensis* NR-03 growth on BEB medium.

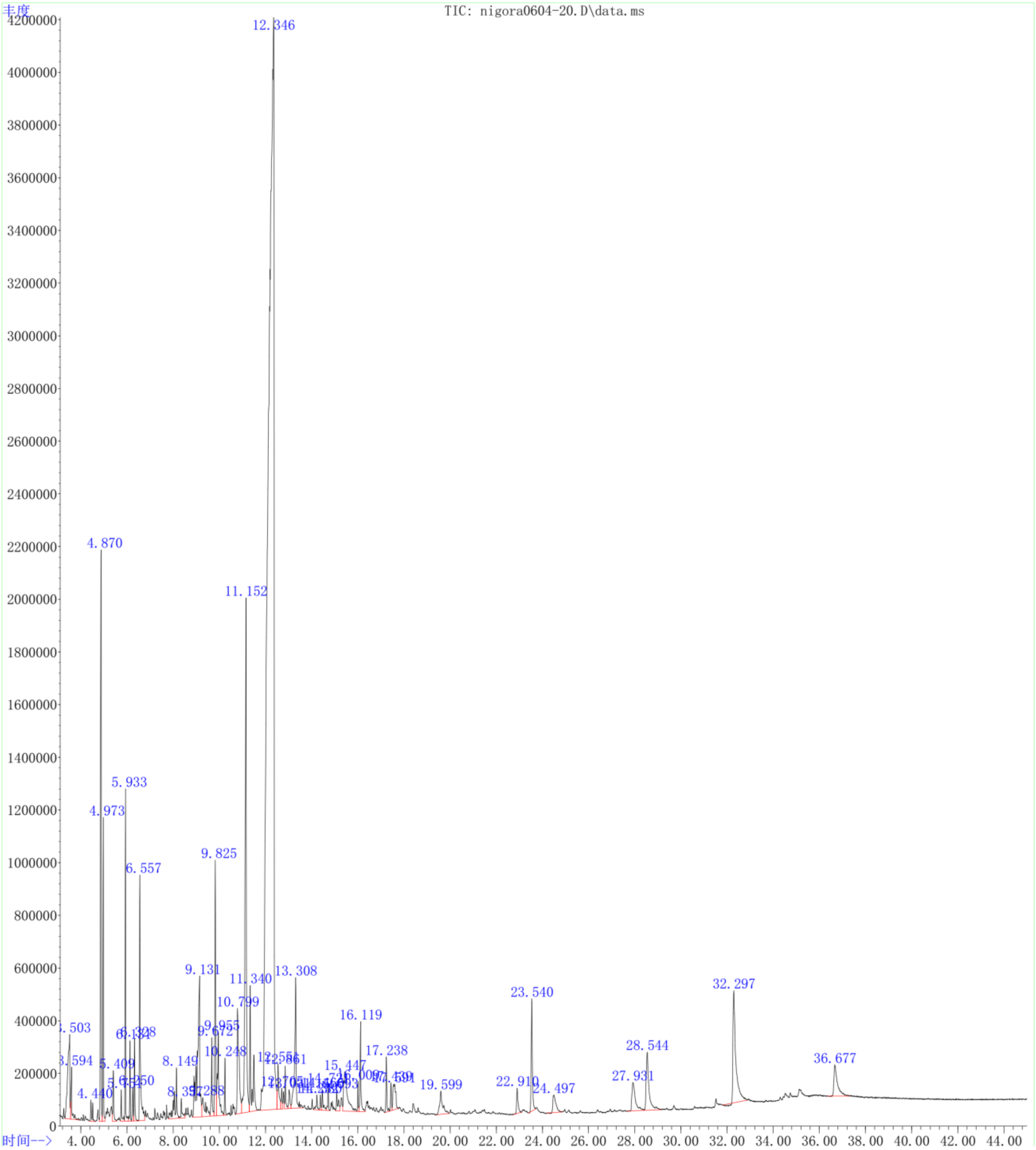

Figure S10. Volatile chemical composition dichloromethane extract of the endophyic fungus of *O. senegalensis* NR-03 growth on BEB medium.

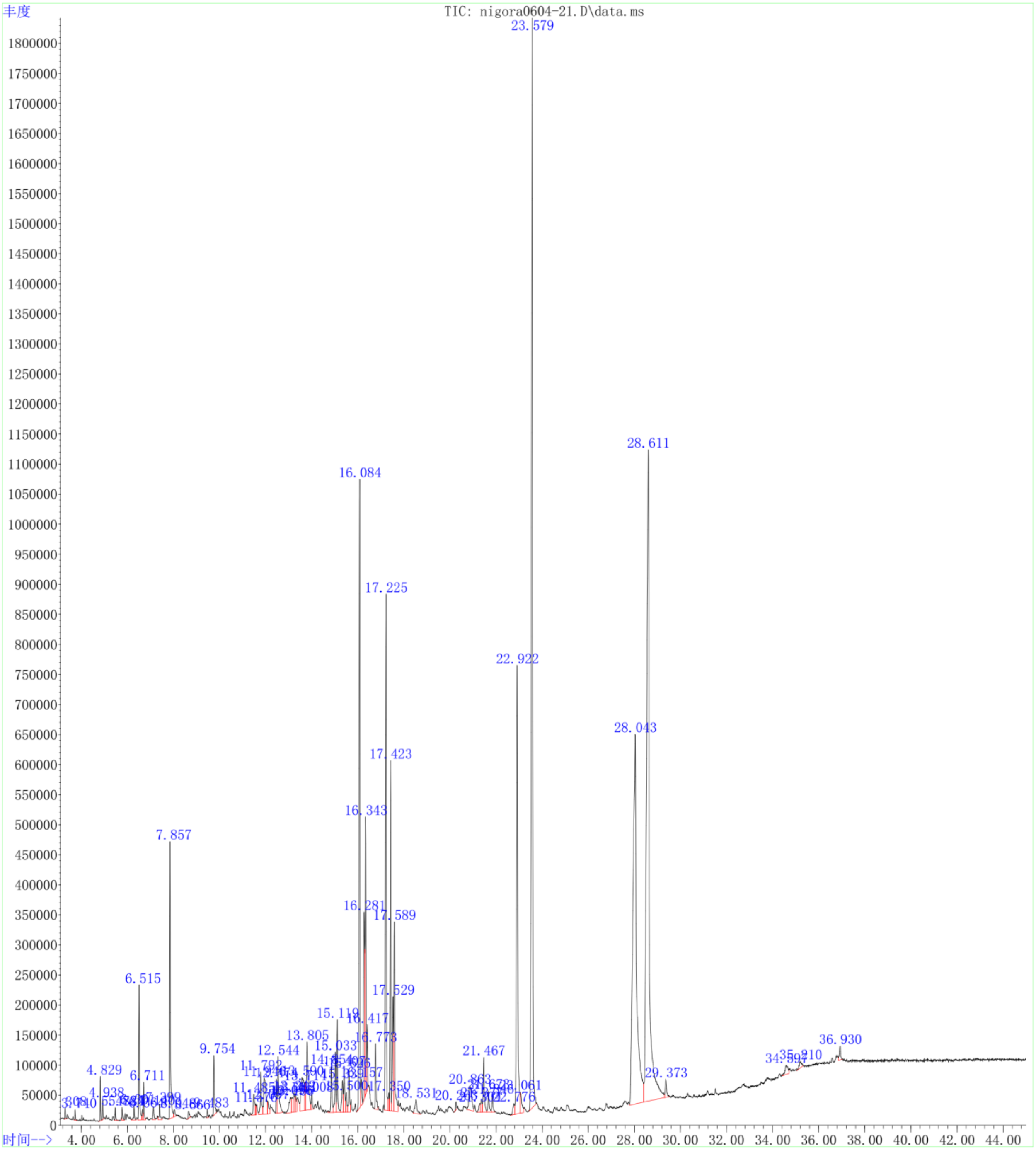

Figure S11. Volatile chemical composition dichloromethane extract of the endophyic fungus of *O. senegalensis* NR-03 growth on YPD medium.

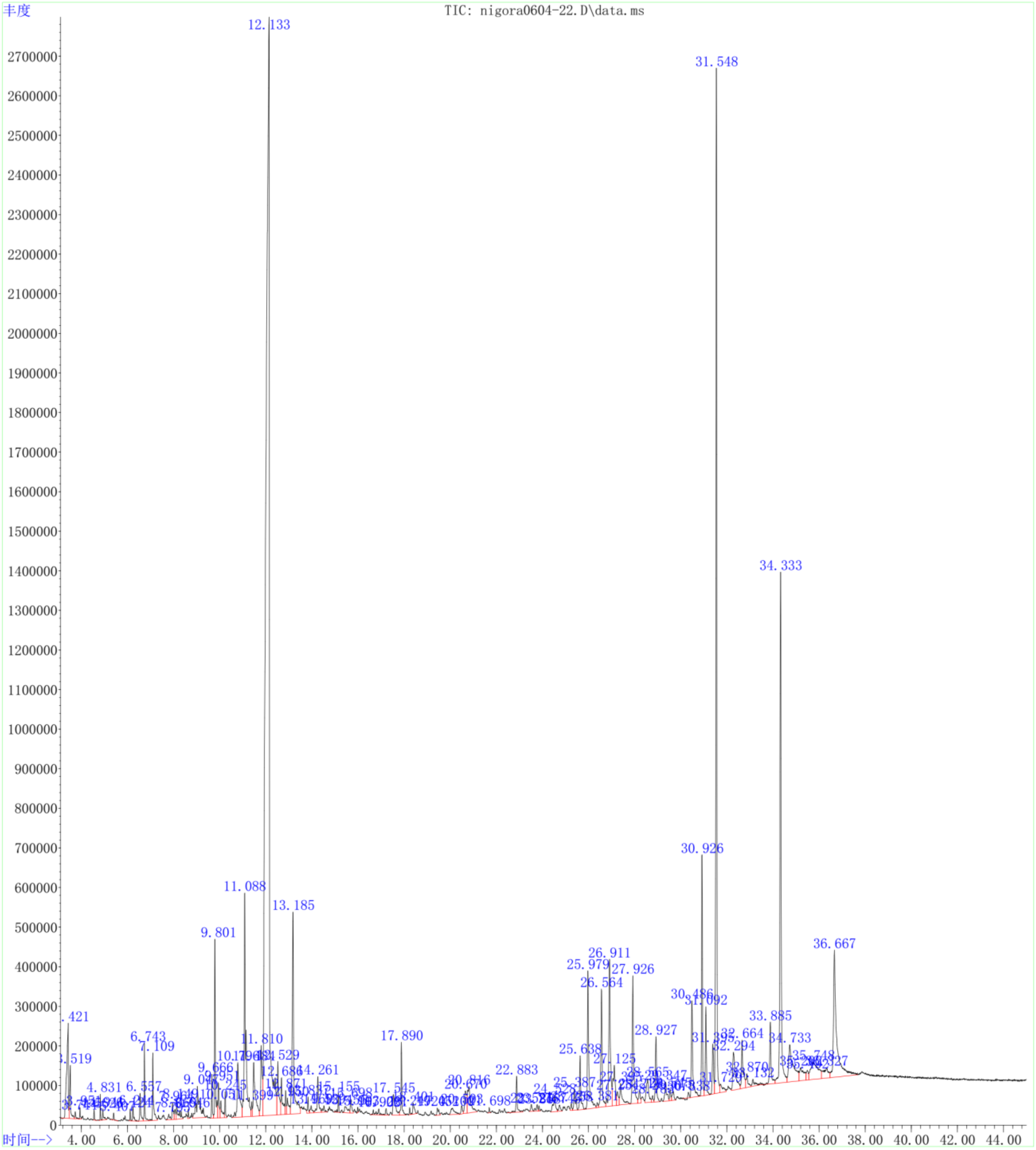

Figure S12. Volatile chemical composition dichloromethane extract of the endophyic fungus of *O. senegalensis* NR-03 growth on MEM medium.

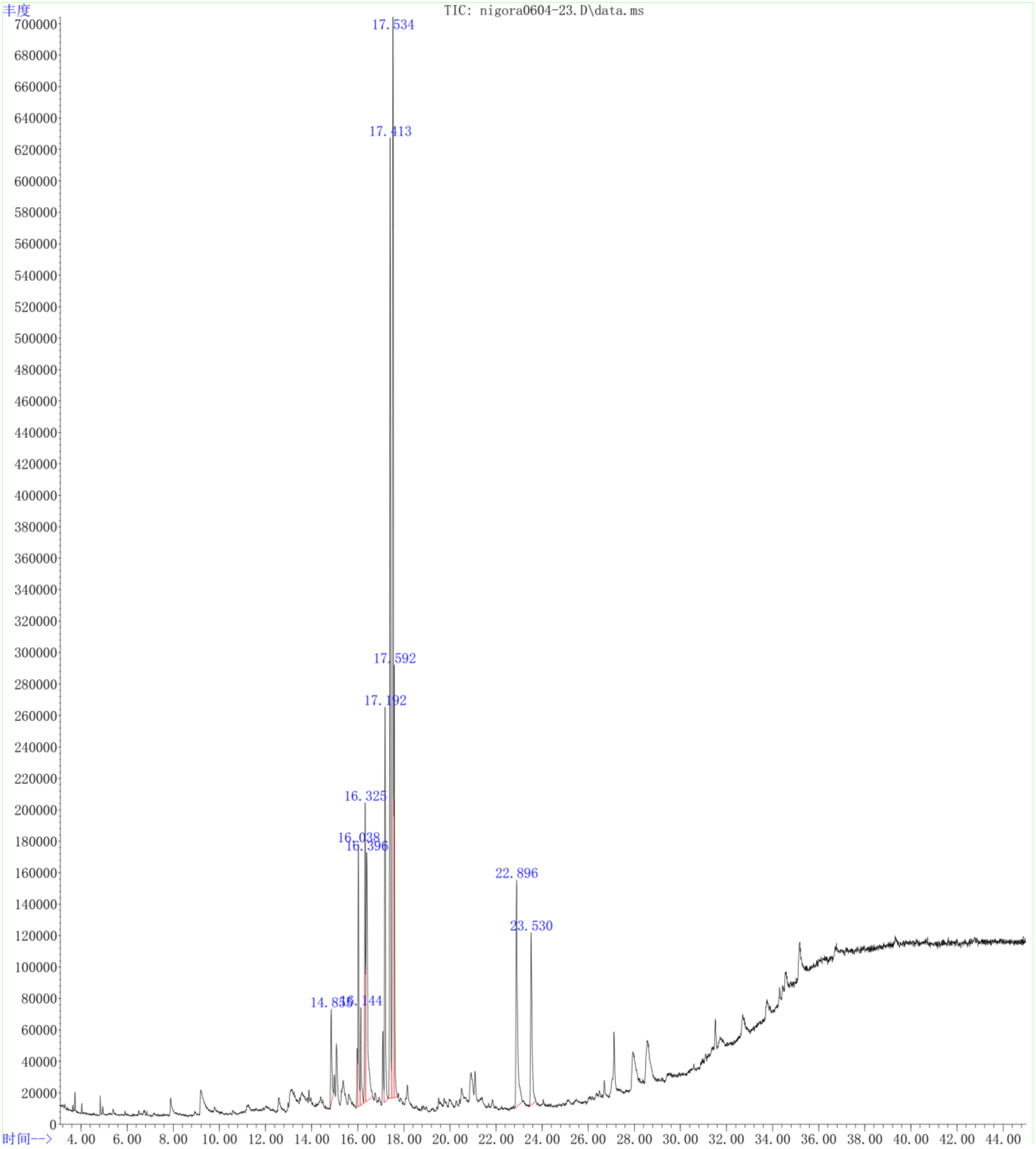

Figure S13. Volatile chemical composition dichloromethane extract of the endophyic fungus of *O. senegalensis* NR-03 growth on PBG medium.

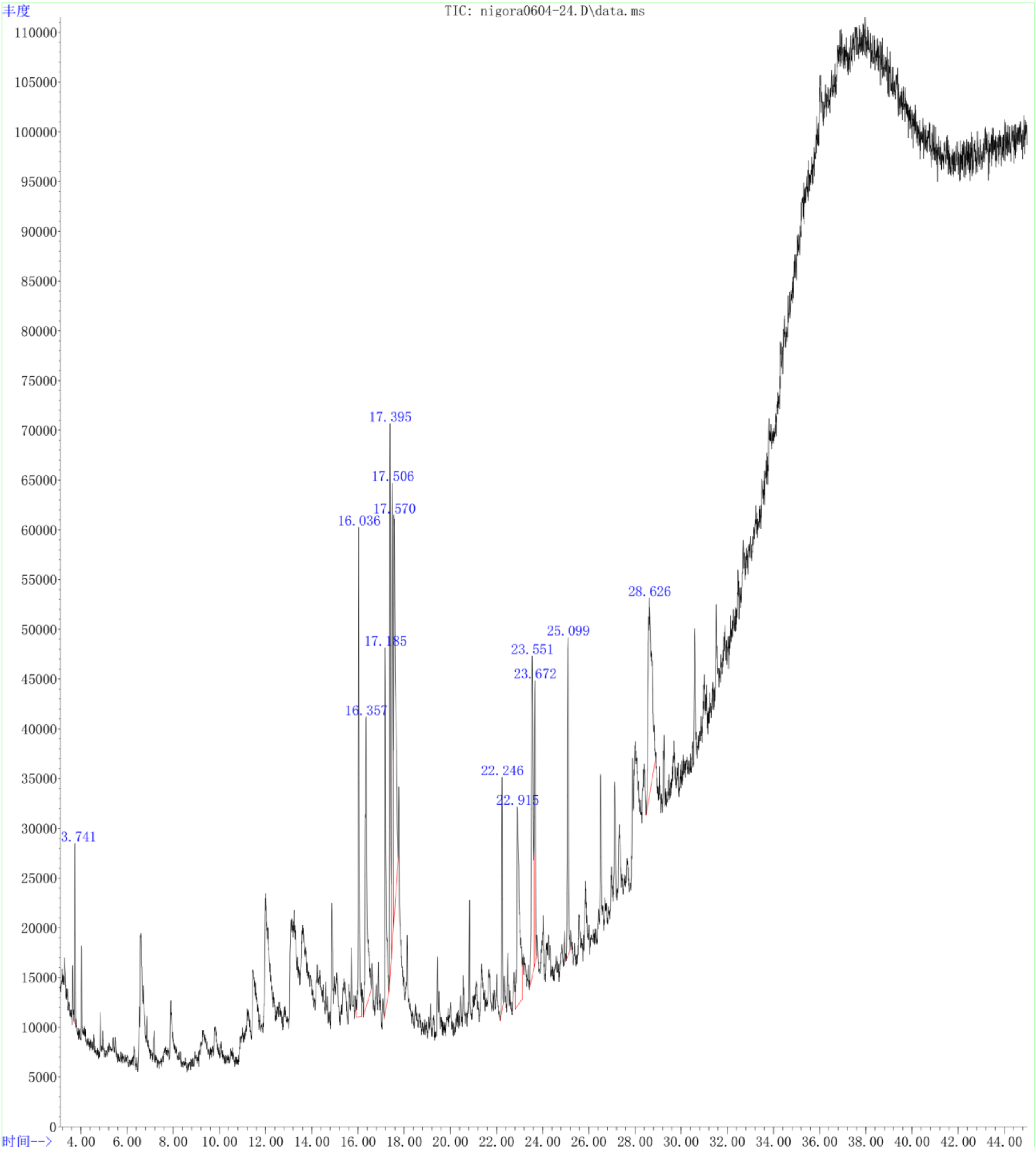

Figure S14. Volatile chemical composition dichloromethane extract of the endophyic fungus of *O. senegalensis* NR-03 growth on CDM medium.

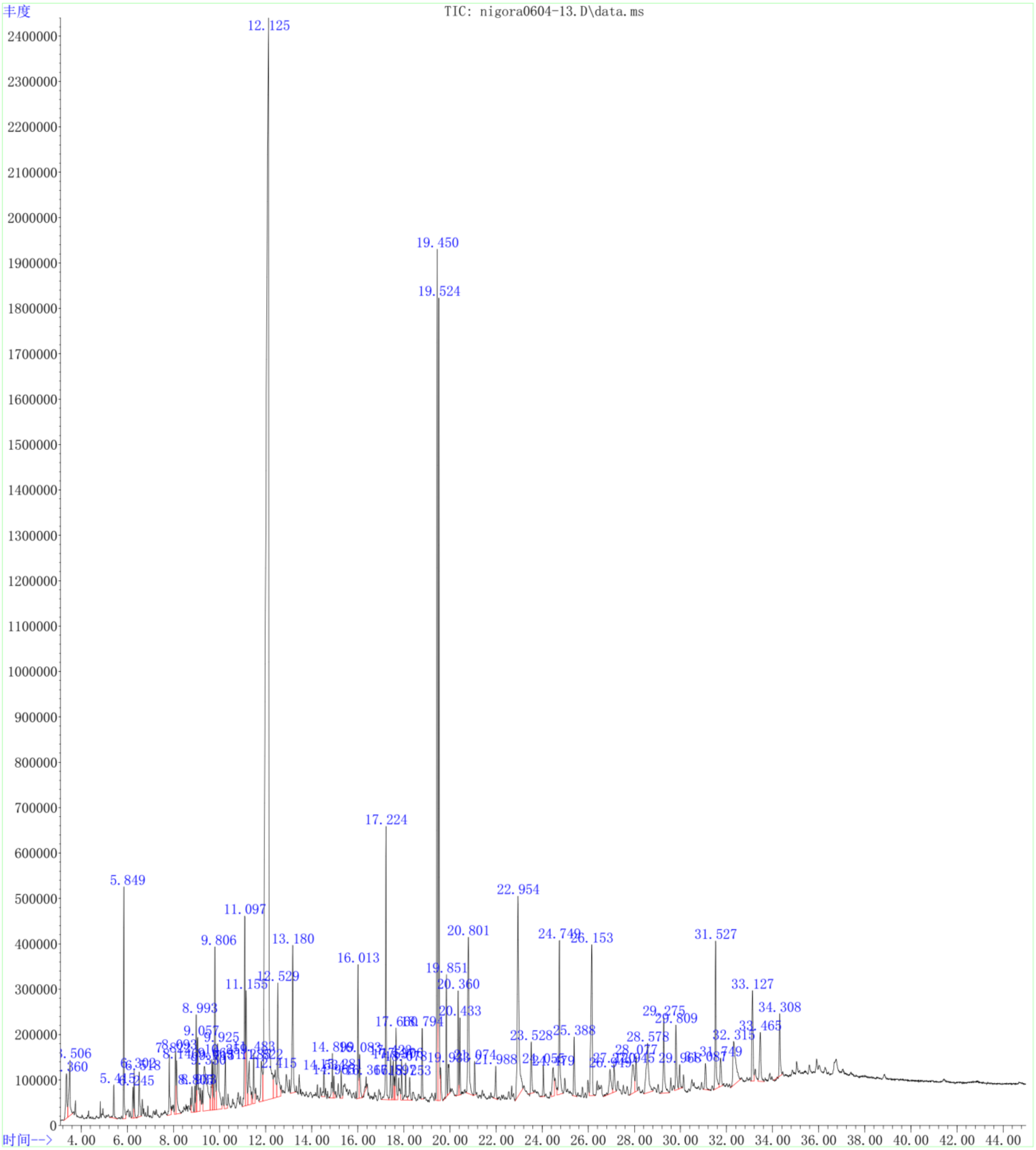

Figure S15. Volatile chemical composition dichloromethane extract of the endophyic fungus of *O. senegalensis* NR-03 growth on PDB medium.

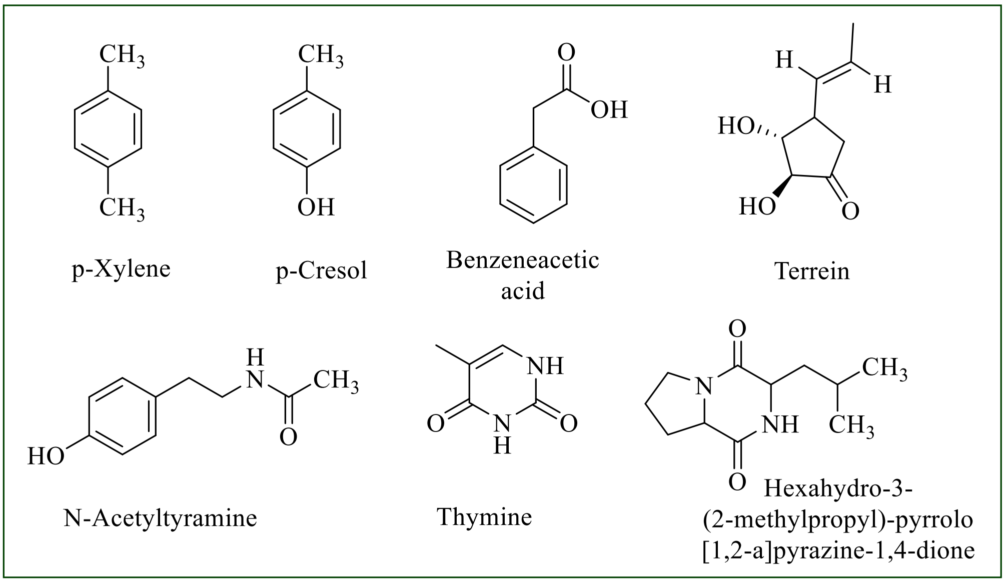

Figure S16. Chemical structure of same main volatile chemical compounds of dichloromethane fraction of *O. senegalensis* NR-03
